# The Role of Calmodulin vs. Synaptotagmin in Exocytosis

**DOI:** 10.3389/fnmol.2021.691363

**Published:** 2021-08-05

**Authors:** Renhao Xue, Hao Meng, Jiaxiang Yin, Jingyao Xia, Zhitao Hu, Huisheng Liu

**Affiliations:** ^1^Shanghai Key Laboratory of Maternal Fetal Medicine, Clinical and Translational Research Center of Shanghai First Maternity & Infant Hospital, School of Life Sciences and Technology, Tongji University, Shanghai, China; ^2^Guangzhou Laboratory, Guangzhou, China; ^3^Bioland Laboratory, Guangzhou Regenerative Medicine and Health Guangdong Laboratory, Guangzhou, China; ^4^Clem Jones Centre for Ageing Dementia Research (CJCADR), Queensland Brain Institute, The University of Queensland, Brisbane, QLD, Australia

**Keywords:** calmodulin, exocytosis, Ca^2+^ sensor, synaptotagmin, vesicles

## Abstract

Exocytosis is a Ca^2+^-regulated process that requires the participation of Ca^2+^ sensors. In the 1980s, two classes of Ca^2+^-binding proteins were proposed as putative Ca^2+^ sensors: EF-hand protein calmodulin, and the C2 domain protein synaptotagmin. In the next few decades, numerous studies determined that in the final stage of membrane fusion triggered by a micromolar boost in the level of Ca^2+^, the low affinity Ca^2+^-binding protein synaptotagmin, especially synaptotagmin 1 and 2, acts as the primary Ca^2+^ sensor, whereas calmodulin is unlikely to be functional due to its high Ca^2+^ affinity. However, in the meantime emerging evidence has revealed that calmodulin is involved in the earlier exocytotic steps prior to fusion, such as vesicle trafficking, docking and priming by acting as a high affinity Ca^2+^ sensor activated at submicromolar level of Ca^2+^. Calmodulin directly interacts with multiple regulatory proteins involved in the regulation of exocytosis, including VAMP, myosin V, Munc13, synapsin, GAP43 and Rab3, and switches on key kinases, such as type II Ca^2+^/calmodulin-dependent protein kinase, to phosphorylate a series of exocytosis regulators, including syntaxin, synapsin, RIM and Ca^2+^ channels. Moreover, calmodulin interacts with synaptotagmin through either direct binding or indirect phosphorylation. In summary, calmodulin and synaptotagmin are Ca^2+^ sensors that play complementary roles throughout the process of exocytosis. In this review, we discuss the complementary roles that calmodulin and synaptotagmin play as Ca^2+^ sensors during exocytosis.

## Introduction

Ca^2+^ is an important signaling molecule that mediates a variety of cellular functions including exocytosis, gene transcription, differentiation, apoptosis, etc. Hence, Ca^2+^-binding proteins are required to serve as sensors to transpose Ca^2+^ signals in these Ca^2+^-dependent processes. A typical Ca^2+^-regulated event is exocytosis, which is a key functional cellular action, as it is responsible for a wide range processes such as the secretion of hormones from endocrine cells, the release of neurotransmitters from presynaptic neurons, the acrosome reaction during fertilization, and the delivery of plasma membrane-bound receptors (Jahn and Sudhof, 1994; Rothman, 1994; Sudhof, 1995, 2012; Wassarman, 1999). During exocytosis, secretory vesicles are directed to release sites at the plasma membrane and become a ready-to-release state through trafficking, docking and priming processes. The well-prepared vesicles subsequently discharge their contents *via* membrane fusion (Lin and Scheller, 2000; Burgoyne and Morgan, 2003). The fusion process is triggered by Ca^2+^ influx and requires the participation of Ca^2+^ sensors. In addition to triggering the final fusion, Ca^2+^ signal is also involved in the steps prior to fusion, indicating that Ca^2+^-binding proteins potentially contribute to earlier stages of vesicle exocytosis (Regehr, 2012).

Two classes of Ca^2+^-binding proteins emerged as Ca^2+^ sensors for exocytosis: EF-hand proteins and C2 domain proteins. A typical EF-hand protein is calmodulin (CaM), which binds Ca^2+^ *via* four EF-hands. CaM is a highly conserved protein ubiquitously and abundantly expressed in eukaryotic cells (Stevens, 1983; Chin and Means, 2000). The C2 domain proteins bind Ca^2+^ *via* the C2 domains that consist of eight β-strands connected by seven loops, two of which normally coordinate calcium ions. The C2 domains have been found in a variety of proteins, such as phospholipase C (PLC), protein kinase C (PKC), and synaptotagmin (Syt).

Since the 1980s, tremendous studies have started seeking Ca^2+^ sensor for exocytosis. At the beginning, researchers focused on both EF-hand and C2 domain proteins because of their Ca^2+^ binding properties. Later studies have found that it is the C2 domain protein Syt, but not the EF-hand protein, acts as the primary Ca^2+^ sensor for membrane fusion. This notion was confirmed by studies in many species, including mouse (Geppert et al., 1994; Nishiki and Augustine, 2004), *Drosophila* (Lee et al., 2013), zebra fish (Wen et al., 2010), as well as the recent studies in the nematode Caenorhabditis *elegans* (Li et al., 2018, 2021). Meanwhile, EF-hand protein CaM, has been found to play an active role in exocytotic steps prior to fusion *via* interaction with multiple functional proteins in a Ca^2+^-dependent manner (Junge et al., 2004; Zikich et al., 2008). Interestingly, CaM and Syt also has crosstalk during exocytosis. In this review, we will summarize the functions of the two types Ca^2+^-binding proteins, CaM and Syt, in membrane fusion and pre-fusion steps to provide a full map describing the role of Ca^2+^ sensors throughout the process of exocytosis.

## Molecular Mechanisms Underlying Dynamic Steps of Exocytosis

**FIGURE 1:**
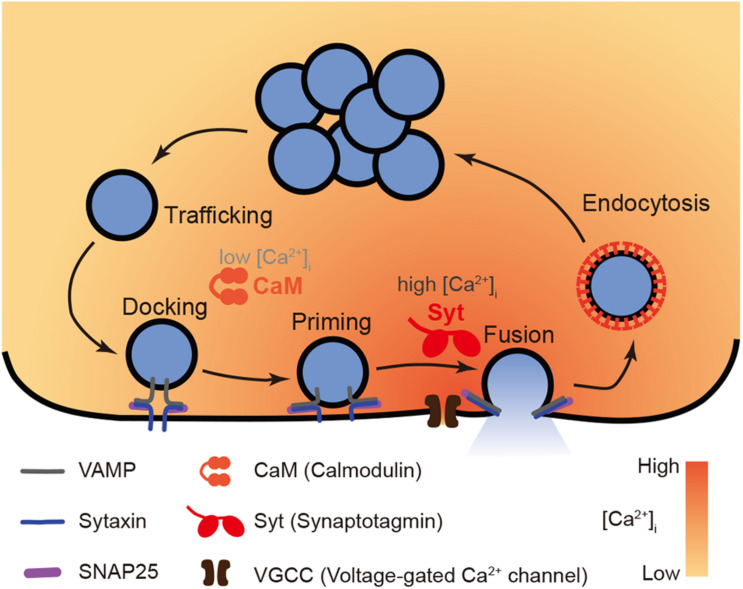
A model of functions of Ca^2+^ sensor CaM vs. Syt in exocytosis. The whole process of Ca^2+^ dependent exocytosis includes vesicle trafficking, docking, priming, and fusion. The final membrane fusion is trigger by Ca^2+^ influx through VGCC that induces a boost of [Ca^2+^]_*i*_ up to micromolar level. The low affinity Ca^2+^ sensor Syt (specifically Syt1 or Syt2) is suitable to convey such high Ca^2+^ signals to vesicle fusion events. Meanwhile, in early steps prior to fusion, the high affinity Ca^2+^ sensor CaM plays a role to facilitate vesicle trafficking, docking, and priming in submicromolar [Ca^2+^]_*i*_.

As described above, exocytosis comprises dynamic steps, including vesicle trafficking, docking, priming, and fusion (Sudhof, 2004; Becherer and Rettig, 2006; Figure 1). Over the past few decades, molecular mechanisms underlying each specific step have been revealed. During trafficking, vesicles wrapped in a lipid bilayer membrane and loaded with secretory cargos, such as hormones or neurotransmitters, are transported from the inner cytosol to the subplasmalemmal region along the microtubule and F-actin tracks by motor proteins (e.g., dynamin and myosin) (Loubery and Coudrier, 2008; Trifaro et al., 2008). Upon arrival, secretory vesicles get tethered to the release sites, and in a following process termed docking, are brought into close contact with the plasma membrane, allowing the vesicle protein VAMP (vesicle-associated membrane protein, also named as synaptobrevin) to interact with the plasma membrane protein syntaxin and the cytoplasmic protein SNAP-25 (synaptosomal-associated protein of 25 kD). Together, VAMP, syntaxin and SNAP-25 can form a protein complex known as the SNARE (soluble N-ethylmaleimide-sensitive factor attachment protein receptor) complex (Sollner, 2003; Verhage and Sorensen, 2008). After docking, vesicles are primed (or mature) in an ATP (adenosine 5’-triphosphate)-dependent manner to a readily releasable state (Chen et al., 2001). Munc13 is known to act as an essential vesicle priming factor (Augustin et al., 1999) that promotes the assembly of SNARE complex by interacting with syntaxin (Betz et al., 1997) and switching it to an active configuration (Ma et al., 2011). Finally, when cells are excited, the boost of [Ca^2+^]_*i*_ (intracellular free Ca^2+^ concentration) triggers the fusion of the vesicle and plasma membrane, and subsequent release of secretory cargos. This membrane fusion is driven by the SNARE complex as a core release machinery (Jahn et al., 2003): the H3 helix of syntaxin interacts with the VAMP coiled-coil domain and two SNAP-25 helices to form coiled-coil bundles. The complex then twists itself to proceed down the energy gradient and bring the lipid bilayers of the vesicle membrane and the plasma membrane sufficiently close to each other to overcome the hydration barrier, and initiate fusion (Burgoyne and Morgan, 2003).

Clearly, membrane fusion, the final step, is triggered by Ca^2+^ signals. As neither v-SNARE (vesicle SNARE: VAMP) nor t-SNAREs (target-SNAREs: syntaxin and SNAP-25) demonstrate direct interactions with Ca^2+^; the final process that releases secretory cargos requires a Ca^2+^ sensor (or Ca^2+^ sensors) to couple the Ca^2+^ signals to the SNARE-driven membrane fusion. Meanwhile, pre-fusion steps are also mediated by a [Ca^2+^]_*i*_ slightly above resting level, although not synchronized with the boost of [Ca^2+^]_*i*_ upon activation. Hence, Ca^2+^-binding proteins might also play roles in secretory vesicle lifespan before the final release.

## The Ca^2+^ Sensor for Membrane Fusion: Syt, Rather Than CaM

Given the critical roles of Ca^2+^ in triggering the final fusion, it must require Ca^2+^ sensor proteins to initiate this step. In this scenario, we will highlight the process of how Syt was identified as the functional Ca^2+^ sensor for fusion, and why CaM was ruled out in this step.

### Why Is CaM Ruled Out as the Ca^2+^ Sensor for Membrane Fusion?

CaM is a common Ca^2+^-binding protein that consists of 148 amino acids (16.7 KDa) and coordinates four calcium ions *via* four EF-hands. Two of these motifs form a globular domain at the N-terminal while the other two constitute an almost symmetrical structure at the C-terminal. A flexible helical linker connects these two globular domains. In response to Ca^2+^ coordinating with the Ca^2+^-binding loops in the EF-hand motifs, a hydrophobic group in each globular domain is exposed, switching CaM to an “open” conformation that is functionally activated (Stevens, 1983; Chin and Means, 2000). CaM is probably the most popular cellular Ca^2+^ binding partner, with diverse functions that vary from buffering intracellular Ca^2+^ to transduction of Ca^2+^ signals. Interestingly, it also interacts with the v-SNARE protein (Quetglas et al., 2000), suggesting a putative role in vesicle exocytosis.

In the 1980s, CaM was shown to be involved in glucose-stimulated insulin release from pancreatic beta cells using a pharmacological inhibitor: trifluoperazine (Krausz et al., 1980; Steinberg et al., 1984). Thereafter, CaM antagonists, including trifluoperazine, W-7 or ophiobolin, were also found to impair catecholamine secretion from bovine chromaffin cells (Clapham and Neher, 1984; Matsumura et al., 1999) and PC12 cells (Quetglas et al., 2002; Ando et al., 2013), inhibit rat parotid amylase exocytosis (Tojyo et al., 1989), affect the mouse acrosome reaction (Bendahmane et al., 2001), diminish neurotransmitter release from various types of nerve terminals (Cazalis et al., 1987; Ando et al., 2013), block the expansion and release of trichocyst matrix during exocytosis in *Paramecium* (Garofalo et al., 1983) and suppress exocytosis in mast cells (Douglas and Nemeth, 1982; Gigl et al., 1987). The involvement of CaM in exocytosis was established in various experimental systems, but two key questions were raised: whether CaM acts as the Ca^2+^ sensor for membrane fusion, and whether CaM actively mediates pre-fusion steps.

The above findings revealed that CaM is a functional mediator of exocytosis. Together with its Ca^2+^-binding properties, CaM was originally speculated to act as a Ca^2+^ sensor for membrane fusion (Brown et al., 1985). However, a clear conclusion cannot be reached simply based on these observations using pharmacological approaches. There are three major arguments against this notion. First, the primary Ca^2+^ sensor for fusion should have an essential effect on vesicle release, with a severe secretion defect being expected if the sensor is blocked. However, inhibition of CaM unlikely induces a strong reduction of release. Second, insufficiency of solid evidence to support the idea that the Ca^2+^-binding activity of CaM is crucial for fusion. Lastly but most importantly, intrinsic Ca^2+^-binding properties make CaM unsuitable to convey Ca^2+^ signals upon [Ca^2+^]_*i*_ boost. Moreover, Syt, another type of Ca^2+^-binding protein, has been found to meet almost all criteria as a primary Ca^2+^ sensor for membrane fusion.

Firstly, the primary Ca^2+^ sensor for membrane fusion should be an essential factor of vesicle release, that it is unlikely for CaM. Indeed, in some research systems for exocytosis, contradictory results have been reported. For example, a CaM antagonist was found to have no effect on Ca^2+^-dependent amylase release from the rat parotid gland (Spearman and Butcher, 1983). In digitonin-permeabilized adrenal chromaffin cells, neither the CaM inhibitor (Wilson and Kirshner, 1983) nor the restoration of reconstituted CaM protein (Sarafian et al., 1987) led to any significant changes in Ca^2+^-evoked secretory activity. Another experiment also revealed dual effects of CaM in transmitter release at neuromuscular junctions (Branisteanu et al., 1989). In mast cells, CaM-mediated disassembly of cortical F-actin was not required for secretion (Sullivan et al., 2000). In human parathyroid adenoma, a CaM inhibitor was found to increase, rather than decrease, parathyroid hormone release (Lu et al., 2011). These findings demonstrated non-identical effect of CaM in different cell types, questioning an essential role for CaM in secretion. It seems like CaM only plays an auxiliary role instead of acting as the primary cellular Ca^2+^ sensing apparatus in the final fusion step.

Secondly, limited direct evidence support that CaM is directly associated with the membrane fusion events in a Ca^2+^-dependent manner. Although the majority of pharmacological studies supported an active role of CaM in exocytosis, mechanism studies were required to elucidate whether it transposes Ca^2+^ signals during fusion. Since the 1990s, with the development of techniques, much more detailed information could be collected from experiments to further elucidate mechanisms underlying observations. Kibble and Burgoyne (1996) measured the rate, instead of quantity of catecholamine release from adrenal chromaffin cells and found that CaM increases the initial release rate, while Watkins and Cooperstein (1997) found using electron microscopy that CaM modulates the interaction between the secretory vesicles and plasma membrane in the parotid gland. These studies began to explore the mechanisms underlying CaM-mediated exocytosis but were not sufficient to draw a final conclusion. It was also reported that CaM functions as a calcium sensitizing factor for cortical granule exocytosis during fertilization of sea urchin eggs, as the Ca^2+^ sensitivity of cortical granule release was largely diminished if CaM was masked by antibodies (Steinhardt and Alderton, 1982). However, a following study denied this conclusion as they claimed that brain extracts failed to restore Ca^2+^ sensitivity in the same system after a brief heat-shock, ruling out the involvement of CaM, given CaM considered to be relatively heat-stable (Sasaki, 1992). Hence, the protein factor that confers Ca^2+^ sensitivity to exocytosis in this system remains an open question. Observation of *Paramecium* using electron microscopy revealed that CaM is essential for the assembly of links that connect the plasma and trichocyst membranes, instead of playing a Ca^2+^-dependent role in stimulus-exocytosis coupling (Kerboeuf et al., 1993). These findings reduced the probability of CaM acting as the membrane fusion Ca^2+^ sensor. However, it should be noted that in some unique cellular systems, it is still possible that CaM is the major Ca^2+^ sensor for fusion. For example, in the fusion of vacuoles (Peters and Mayer, 1998) and sperm-specific membranous organelle (Shang et al., 2013).

Finally, Burgoyne and Clague (2003) pointed out that CaM is a relatively high affinity Ca^2+^ sensor that is fully activated at submicromolar [Ca^2+^]_*i*_ (∼0.5 μM). This Ca^2+^ concentration is much lower than the boost of [Ca^2+^]_*i*_ in most activated cells (e.g., neurons), which is above the micromolar level. If CaM serves as the Ca^2+^ sensor, membrane fusion would theoretically occur even in the absence of the [Ca^2+^]_*i*_ boost. Therefore, the real Ca^2+^ sensor that triggers exocytotic membrane fusion should have a lower affinity to Ca^2+^ than CaM.

### How Is Syt Identified as the Ca^2+^ Sensor for Membrane Fusion?

Although considerable attention was driven to CaM, a significant body of research elucidated that the other class of putative Ca^2+^ sensor, Syt, is indeed responsible for the transit of Ca^2+^ signals to membrane fusion. Syt is a family of C2 domain-containing proteins with 17 isoforms (Craxton, 2004). Most of Syt isoforms consist of two C2 domains. The best studied isoform is Syt1 (synaptotagmin I), which is a membrane protein that binds with Ca^2+^ through its tandem C2 domains (C2A and C2B) with a much lower affinity than that of CaM (K_*d*_ > 10 μM) (Shao et al., 1998; Fernandez-Chacon et al., 2001). Hence, this Ca^2+^ sensor can only be switched on by robust Ca^2+^ signals such as the Ca^2+^ influx triggered by action potentials at presynaptic nerve terminals. Syt also binds with both t-SNARE proteins [syntaxin (Bennett et al., 1992) and SNAP25 (Schiavo et al., 1997; Zhou et al., 2015)], and these interactions are Ca^2+^ dependent (Chapman et al., 1995; Gerona et al., 2000). This intrinsic property makes Syt1 a perfect candidate of Ca^2+^ sensor for membrane fusion in the final step of exocytosis.

Unlike CaM, considerable evidence supports the role of Syt as major Ca^2+^ sensors for membrane fusion. In the 1990s, various studies revealed that inactivation of Syt by mutation, inhibitory peptide or knockout, largely impaired neurotransmitter release in the nematode *C. elegans* (Nonet et al., 1993), *Drosophila* (Littleton et al., 1993), squid *Loligo pealei* (Bommert et al., 1993), and mouse (Geppert et al., 1994), suggesting an essential role of Syt conservative through the evolution. Early in this century, advanced electrophysiological technique together with molecular biological approaches allowed researchers to modify proteins *via* various mutations and measure the release rate of synaptic vesicles driven by modified proteins. It was found that changes in the Ca^2+^ coordination activity of Syt *via* Ca^2+^ ligand mutants in the C2 domains lead to dramatic changes in the rate of release and its cooperativity with the extracellular Ca^2+^ concentration (Fernandez-Chacon et al., 2001; Mackler et al., 2002; Robinson et al., 2002; Stevens and Sullivan, 2003; Nishiki and Augustine, 2004). These findings provided direct evidence that the Ca^2+^-binding activity of Syt involves membrane fusion. Despite this, doubts remained regarding whether Syt simply synchronize vesicle release *via* interactions with voltage-gated Ca^2+^ channels (VGCC) and therefore does not indeed convey Ca^2+^ signals to release events (Neher and Penner, 1994). To answer this question, studies using flash photolysis of caged Ca^2+^ as Ca^2+^ source to bypass Ca^2+^ channels ruled out this possibility (Sun et al., 2007; Burgalossi et al., 2010). These studies confirmed Syt as the major Ca^2+^ sensor for fusion. Syt1 and Syt2 (synaptotagmin 2) are the two Syt isoforms that demonstrate the lowest Ca^2+^ affinity (Sugita et al., 2002; Pinheiro et al., 2016). These isoforms are also functional in neurons which exhibit an enormous and sharp [Ca^2+^]_*i*_ peak when excited. Syt1 is active in hippocampal excitatory neurons (Geppert et al., 1994) while Syt2 is functional in cortical inhibitory neurons (Chen et al., 2017). In the case of endocrinal cells, Syt1 and Syt7 (synaptotagmin 7) are overlapping Ca^2+^ sensors that both trigger large dense core vesicle (LDCV) fusion in chromaffin cells (Voets et al., 2001; Schonn et al., 2008). Syt7 is responsible for insulin vesicle fusion in pancreatic beta cells (Gustavsson et al., 2008). These findings suggested a general role of Syt as a Ca^2+^ sensor in a variety of cell types. Moreover, when Syt1 was chemico-genetically engineered to sense Sr^2+^, a non-physiological metal, Sr^2+^-dependent exocytosis was observed in cultured neurons, confirming that Syt1 acts as a functional metal sensor for release (Evans et al., 2015). Together, these lines of evidence support the idea that Syt functions as the primary Ca^2+^ sensor, driving the Ca^2+^-evoked fusion events of synaptic vesicles (Figure 1).

### CaM Is Unlikely the Ca^2+^ Sensor for Membrane Fusion Even at Low [Ca^2+^]_*i*_

As described above, CaM is not a Ca^2+^ sensor for final fusion due to its high Ca^2+^ affinity. Nonetheless, it should be noted that the final release event does not always coincident with a [Ca^2+^]_*i*_ boost above the micromolar level, but sometimes also occurs at submicromolar [Ca^2+^]_*i*_. For example, in neurons, asynchronous release of synaptic vesicles does not synchronize with the peak of Ca^2+^ signal, but accompanies a post-peak [Ca^2+^]_*i*_ which is only a little above the resting [Ca^2+^]_*i*_. Moreover, secretion of neurotransmitters can even occur spontaneously at a [Ca^2+^]_*i*_ close to basal level. Hence, it is still questionable whether CaM senses Ca^2+^ for fusion at low [Ca^2+^]_*i*_ during asynchronous or spontaneous exocytosis.

An electrophysiological study that measured the release rate and [Ca^2+^]_*i*_ simultaneously suggested an allosteric model for the release Ca^2+^ sensor (Lou et al., 2005). According to this model, Ca^2+^ sensors that mediate release at distinct [Ca^2+^]_*i*_ are structurally distinct. Hence, it is likely that different Ca^2+^ sensors with different Ca^2+^ affinities mediate different types of release. Using the same method, it was found that removal of Syt2, the Syt with low Ca^2+^ affinity, affects the release rate only at high [Ca^2+^]_*i*_, but not at submicromolar [Ca^2+^]_*i*_, in the calyx of Held (Sun et al., 2007). Consistently, knockout of Syt1 or Syt2, the two synaptotagmins with low Ca^2+^ affinity, only eliminated synchronous neurotransmitter release, without reducing (actually, even increasing) asynchronous or spontaneous release (Geppert et al., 1994; Liu et al., 2009). This led to the conclusion that Syt1 and Syt2 were Ca^2+^ sensors only for synchronous vesicle fusion in neurons.

As a high affinity Ca^2+^ sensor, CaM is plausibly suitable for triggering asynchronous or spontaneous synaptic vesicle release. However, the fact that knockdown of CaM failed to reduce asynchronous neurotransmitter release in Syt1 knockout cortical neurons (Pang et al., 2010b), directly contradicted this idea. Evidence subsequently emerged to indicate that Syt7, a high Ca^2+^ affinity Syt isoform (Sugita et al., 2002) might act as a Ca^2+^ sensor for asynchronous release (Bacaj et al., 2013) and double C2-like domain-containing protein (Doc2), another C2 domain protein with relatively high Ca^2+^ affinity, senses Ca^2+^ signal in both asynchronous (Yao et al., 2011; Xue et al., 2015) and spontaneous release (Groffen et al., 2010). Hence, CaM is unlikely to act as a Ca^2+^ sensor that directly triggers membrane fusion. Nevertheless, it is still possible that it plays a secondary role by mediating key proteins, such as Syt.

## CaM Plays Ca^2+^-Dependent Roles in Pre-Fusion Steps

The early processes of vesicle exocytosis, namely trafficking, docking and priming are crucial preparations for the final release of the contents of vesicles, but are not associated with the peak of the [Ca^2+^]_*i*_ boost upon cell excitation. Hence, low affinity Ca^2+^ sensors, such as Syt1 and Syt2, are not activated, whereas high affinity Ca^2+^ sensors, i.e., CaM, might be functionally involved in these pre-fusion steps due to a [Ca^2+^]_*i*_ below the micromolar level (Burgoyne and Clague, 2003).

Through these early steps, secretory vesicles are delivered to the release sites and conveyed into a mature state ready for release. These mature vesicles constitute a readily releasable pool (RRP) (Rizzoli and Betz, 2005). The maintenance of the RRP before release and the subsequent replenishment of this pool after release are crucial determinants of the extent of final release. Using a pharmacological inhibitor, it was found that CaM is involved in the delivery of vesicle into the RRP in the calyx of Held (Sakaba and Neher, 2001) and hippocampal neurons (Liu H. et al., 2014). CaM also mediates short-term synaptic plasticity at least partly through the recovery of RRP (Junge et al., 2004; Lipstein et al., 2013). It has also been reported that CaM supports the recovery from short-term depression at retinal cone ribbon synapses, but the mechanisms underlying this acceleration and its functional implications for vision are unknown (Van Hook et al., 2014). In summary, CaM clearly plays a role in exocytotic actions prior to final vesicular fusion (Figure 1).

How does CaM complete its role? Once activated by Ca^2+^ binding, CaM may mediate the function of multiple key regulatory exocytotic proteins through direct interactions or modulation by phosphorylation *via* activation of some important kinases (Creutz et al., 1983; Watkins and White, 1985).

### CaM Direct Interactions With Key Regulatory Proteins in Pre-fusion Steps

CaM may contribute to the early steps of exocytosis *via* direct binding with components of the SNARE complex. As described above, the SNARE complex, consisting of v-SNARE VAMP, and t-SNARE SNAP25 and syntaxin, serves as the core machinery of membrane fusion. It has been reported that CaM binds to VAMP2, the v-SNARE, but not to syntaxin or SNAP25, the t-SNAREs, and that this direct binding mediates the lipid interaction activity of VAMP2 (Quetglas et al., 2000). If this binding is diminished, the formation of the SNARE complex is impaired and hence the hormone secretion from chromaffin cells is inhibited (Quetglas et al., 2002). Another study also revealed that Ca^2+^/calmodulin mediates SNARE assembly by transferring VAMP from cis-membrane to trans-membrane (de Haro et al., 2004). These findings suggested that CaM is an active mediator of secretory vesicle docking and priming *via* interaction with of v-SNARE.

The interaction partners of CaM are not limited to the SNARE proteins. Another important binding partner of CaM is myosin V (Espindola et al., 1992). Myosin-V is a key molecular motor that drives vesicle trafficking along F-actin (Reck-Peterson et al., 2000). In the presence of CaM and Ca^2+^, syntaxin-VAMP-myosin V form a complex (Ohyama et al., 2001). At the switch point where vesicles are unloaded from cytoskeleton tracks and tethered to the plasma membrane, submicromolar [Ca^2+^]_*i*_ releases CaM from myosin V, the syntaxin-1A interaction site in myosin V is exposed. Then syntaxin-1A binds with myosin V and consequently vesicle docking is completed (Watanabe et al., 2005). This CaM-myosin interaction underscores the role of CaM during trafficking and docking.

Munc13, which plays an essential role in vesicle priming (Augustin et al., 1999), also exhibits robust binding property with CaM. The two major Munc13 isoforms, Munc13-1 and ubMunc13-2, bind with CaM in the region between the first C2 domain and the C1 domain. Active mutants at this region which eliminate CaM binding activity also impair short-term plasticity, at least partly by inhibiting the replenishment of the RRP (Junge et al., 2004). These results reveal a key role of CaM-Munc13 binding in Munc13-induced vesicle priming. A similar functional interaction has also been observed in chromaffin cells (Zikich et al., 2008). UNC-13, the homolog of Munc13 in *C. elegans*, also binds with CaM to accelerate the release (Hu et al., 2013). These findings reveal a crucial mechanism underlying CaM-mediated vesicle priming.

**FIGURE 2:**
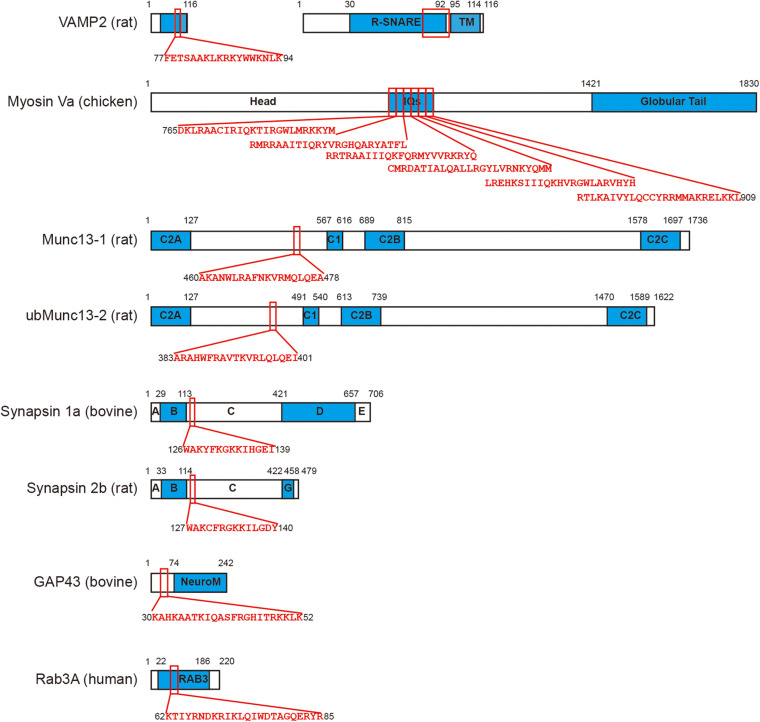
CaM directly binds with a series of functional proteins in exocytosis. VAMP2 (Quetglas et al., 2000), myosin Va (Espindola et al., 1992), Munc13-1, ubMunc13-2 (Junge et al., 2004), synapsin 1a, synapsin 2b (Nicol et al., 1997), GAP43 (Baudier et al., 1991), and Rab3A (Park et al., 1997) are all binding partners of CaM. The CaM binding motifs in each protein are labeled using red squares and the corresponding sequence of amino acid is shown. Please note myosin Va consists of six tandem IQ motifs that interact with CaM. Since the size of VAMP2 is relatively small, a 5-times amplification is displayed on its right.

CaM also interacts with synapsin, GAP43, Rab3, and Syt. Synapsin is a functional protein that is involved in synaptic vesicle trafficking, docking, and release in presynaptic nerve terminals and modulates synaptic plasticity (Greengard et al., 1993; Cesca et al., 2010). CaM directly binds to synapsin 1 (Hayes et al., 1991) and synapsin 2 (Nicol et al., 1997), the two major isoforms of synapsin family. GAP43 is a regulator of exocytosis that directly interacts with the core complex of exocytosis and Syt (Haruta et al., 1997). CaM binding with GAP43 (Baudier et al., 1991) may impair the priming step by inhibiting phosphorylation of GAP43 (Misonou et al., 1998). Rab3 is a small GTPase that is involved in exocytosis as an inhibitory regulator (Johannes et al., 1994; Geppert et al., 1997). CaM binds with Rab3 (Park et al., 1997) and such interaction was found to abolish the Rab3-induced inhibition of vesicle release from catecholamine and insulin secreting cells (Coppola et al., 1999), but not in PC12 cell (Schluter et al., 2002). CaM also promotes GTP binding to Rab3A (Park et al., 1997), forming an active GTP-bound Rab3A-Ca^2+^/CaM complex (Park et al., 2002), and the Rab3A-CaM interaction involved in insulin secretion from pancreatic beta cells (Kajio et al., 2001), and acrosome exocytosis (Yunes et al., 2002). In summary, CaM directly interacts with a variety of functional proteins, usually in a Ca^2+^-dependent manner, to regulate early steps of exocytosis (Figure 2).

### CaM Induces Phosphorylation of Regulatory Proteins in Pre-fusion Steps by Activating Key Kinases

Some of the key functional proteins in exocytosis are substrates of kinases that need to be activated by CaM (Cooperstein and Watkins, 1995). A typical kinase related to CaM is type II Ca^2+^/CaM dependent protein kinase (CaMKII). CaMKII (Colbran, 1992) is important for exocytosis (Ammala et al., 1993), including GABA release at inhibitory synapses (Sitges et al., 1995), insulin release from pancreatic beta cells (Ashcroft et al., 1994), catecholamine release from PC12 cells (Schweitzer et al., 1995), calcium-evoked dendritic exocytosis (Maletic-Savatic et al., 1998), postsynaptic secretion of BDNF (brain-derived neurotrophic fact) and neurotrophin-3 from hippocampal neurons (Kolarow et al., 2007), acetylcholine and GABA release at the *C. elegans* neuromuscular junctions (Liu et al., 2007), and neurotransmitter release in cultured neurons (Pang et al., 2010a). CaMKII also contributes to plasma membrane resealing in response to cell membrane injury, by facilitating membrane fusion through vesicle exocytosis (Steinhardt et al., 1994). Activated CaMKII phosphorylates multiple proteins to switch on their functions in regulation of exocytosis.

Interestingly, the most important substrate of CaMKII is itself. Twelve CaMKII molecules usually form a functional dodecamer. Upon interaction with CaM in the presence of Ca^2+^, the CaMKII dodecamers undergo autophosphorylation and become activated (Miller and Kennedy, 1986; Meyer et al., 1992; Hanson et al., 1994). Such autophosphorylation has been found to mediate the plasticity of synaptic vesicle release (Giese et al., 1998). CaMKII directly binds to syntaxin 1A at plasma membrane in a Ca^2+^-and ATP-dependent manner, with autophosphorylation of CaMKII largely facilitating this interaction (Ohyama et al., 2002; Nomura et al., 2003; Watanabe et al., 2013). These CaMKII-syntaxin-1A interactions have been found to be important for regulated exocytosis (Ohyama et al., 2002).

Some SNARE proteins are also substrates of CaMKII. Although there is no evidence to indicate that syntaxin-1A is directly phosphorylated by CaMKII, syntaxin-3, another isoform of syntaxin, was identified as a substrate of CaMKII (Risinger and Bennett, 1999). In ribbon synapses of the retina, CaMKII-induced phosphorylation of syntaxin-3B mediated the exocytosis of synaptic vesicles by modulating the assembly of the SNARE complex (Liu X. et al., 2014). Furthermore, CaMKII can also phosphorylate the v-SNARE protein VAMP (Nielander et al., 1995).

Besides the autophosphorylation and phosphorylation of SNARE proteins, CaMKII also phosphorylates a variety of regulatory exocytotic proteins, such as synapsin (Baines and Bennett, 1985), Rab3-interacting molecule (RIM) (Sun et al., 2003), Ca^2+^ channels (Sun et al., 2003), and even Syt (Popoli, 1993; Hilfiker et al., 1999). For example, RIM is an important mediator of synaptic vesicle release (Wang et al., 1997; Schoch et al., 2002; Deng et al., 2011). Its serine sites are phosphorylated by Ca^2+^/CaM, which promotes the interaction of 14-3-3 with the N-terminal of RIM (Sun et al., 2003). CaMKII also phosphorylates the voltage-gated Ca^2+^ channels, Ca_*v*_2.1 and Ca_*v*_2.2, and subsequently modulates their interaction with SNARE proteins (Yokoyama et al., 2005).

Moreover, Ca^2+^/CaM dependent kinases are not limited to CaMKII. There are still other kinases that are functionally involved in exocytosis in a Ca^2+^-and CaM-dependent manner, such as myosin light chain kinase (MLCK). This kinase was found to play a role in thyroid hormone secretion *via* phosphorylation of the myosin light chain (Tawata et al., 1984). It also mediates the release of secretory granules from the pituitary gland (Nelson et al., 1987), and exocytosis of amylase from the parotid gland (Hashioka and Kato, 1990). Another study using bovine chromaffin cells established the role of MLCK in the priming of secretory vesicles (Matsumura et al., 1999). MLCK also enhances the size of the RRP without affecting the release probability in post-tetanic potentiated calyx of Held synapses (Lee et al., 2008). This is consistent with the idea that CaM is mainly involved in pre-fusion steps of exocytosis.

### Concurrent Effects of Direct Interaction and Phosphorylation Modification

As described above, CaM can either directly interact with or indirectly phosphorylate (*via* CaM-dependent kinases) multiple regulatory proteins that are important for exocytosis. Notably, such direct interaction and indirect phosphorylation are not always isolated events. A good example is synapsin I. In order to play its role in exocytosis, synapsin I must be phosphorylated and it also serves as a substrate of CaMKII (Baines and Bennett, 1985). Interestingly, this phosphorylation is dependent on Ca^2+^-dependent binding of CaM with the head domain of synapsin 1 (Hayes et al., 1991; Goold and Baines, 1994). Upon CaM-synapsin I binding, CaMKII is activated and phosphorylates the synapsin I. This in turn mediates the interactions between synapsin I and cytoskeletal proteins or small synaptic vesicles (Petrucci et al., 1991), and mobilizes synaptic vesicles (Chi et al., 2003), thereby playing a role in exocytosis, such as facilitating synapses if dynamin-dependent vesicle recycling is impaired (Lou et al., 2012), mediating insulin vesicle release (Yamamoto et al., 2003), or modulating fusion pore kinetics of dense core vesicles in PC12 cells (Yang et al., 2021). In this situation, binding with CaM does not directly activate the protein but induces its phosphorylation *via* a CaM-dependent kinase and subsequent functionalization. Clearly, these two mechanisms are highly correlative. Besides synapsin, there are other proteins that are both binding partners of CaM and substrates of CaM-dependent kinase, such as VAMP and Syt. These proteins can be modulated by CaM through both mechanisms. However, it is still unclear whether these two mechanisms act synergistically or independently for each individual protein.

It should be noted that, in addition to direct binding and indirect phosphorylation, CaM is also involved in other cellular processes under physiological conditions. For instance, CaM enhances ribbon replenishment and shapes filtering of synaptic transmission by functioning as an endogenous buffer of intracellular Ca^2+^ (Van Hook et al., 2014). It has also been reported that CaM plays a dominant role in inhibiting vesicular release and modulating short-term synaptic plasticity (Timofeeva and Volynski, 2015).

In summary, CaM is an active mediator of exocytosis that is mainly involved in vesicle trafficking, docking and priming steps prior to membrane fusion. The major mechanism underlying this function is modulation of key regulatory proteins through Ca^2+^-dependent direct binding and phosphorylation *via* Ca^2+^/CaM activated-kinases. In general, a submicromolar [Ca^2+^]_*i*_ level is required for CaM to act. Hence, CaM is considered as a high affinity Ca^2+^ sensor for the pre-fusion steps, while Syt is a low affinity Ca^2+^ sensor for the final fusion (Figure 1).

## Interactions Between CaM and Syt

Finally, it is worth paying some attention to the direct interplay between the two major classes of Ca^2+^-binding proteins. CaM, the EF-hand protein, can interact with Syt, the C2 domain protein. Hence, to some extent, the function of the Ca^2+^ sensor in membrane fusion might also be modulated by CaM. Perin (1996) demonstrated that multiple isoforms of Syt bind with CaM *via* a neuroxin-binding region close to the C-terminal. However, the physiological function of this interaction remained unknown until recent report that CaM-Syt7 binding is essential for the Syt7-mediated refilling of the RRP during synaptic depression (Liu H. et al., 2014) and the replenishment of insulin vesicle pool in pancreatic beta cells (Dolai et al., 2016). These findings reinforced an important role of CaM in vesicle trafficking, docking and priming, but it still remains unclear whether the EF-hands in CaM or the C2 domains in Syt7 are the major active Ca^2+^ sensing modules in the CaM-Syt7 complex.

In addition to direct binding, it has been reported that Syt can be phosphorylated by CaMKII, which is activated by Ca^2+^-bound CaM (Popoli, 1993; Hilfiker et al., 1999; Celano et al., 2003). This phosphorylation facilitates its Ca^2+^-dependent interaction with t-SNAREs (Verona et al., 2000). In light of these observations, CaM is highly likely to be involved in the modulation of Syt’s function during exocytosis *via* either direct binding or indirect phosphorylation. It is still unknown whether the indirect phosphorylation is dependent on the direct binding, as for synapsin. Future studies are required to address this question.

CaM and Syt can also inhibit each other. It has been reported that Ca^2+^/CaM suppresses the expression of Syt2 in cortical neurons *via* an unknown mechanism (Pang et al., 2010b). In an *in vitro* liposome fusion assay, CaM was also found to inhibit, rather than facilitate, membrane fusion in the absence of Syt, with these inhibitory effects being abolished in the presence of Syt (Di Giovanni et al., 2010). These studies demonstrate that the crosstalk between CaM and Syt is complicated. Further studies are therefore required to fully elucidate the mechanism and function of this remarkable protein-protein interaction.

## Conclusion and Perspective

In conclusion, during exocytosis, Syt, but not CaM, senses Ca^2+^ signals and consequently triggers the final fusion events. Although not the Ca^2+^ sensor for fusion, CaM might act as a putative high affinity Ca^2+^ sensor that is switched on at low [Ca^2+^]_*i*_, which is close to resting [Ca^2+^]_*i*_, thereby working in temporal disassociation from the [Ca^2+^]_*i*_ boost or spatially distant to the Ca^2+^ micro/nano domain. Hence, it mainly mediates early exocytotic steps prior to membrane fusion, such as vesicle trafficking, docking and priming, *via* direct interactions with and/or indirect phosphorylation of key regulatory exocytotic proteins.

To further understand the roles of Ca^2+^-binding proteins CaM during exocytosis, several key questions need to be addressed in future studies. Does CaM play a secondary role in final membrane fusion? What is the impact of the CaM-Syt interaction in exocytosis? Are any interaction partners of CaM or substrates of CaM-dependent kinases still to be identified? Addressing these questions will enhance our understanding of the entire process of exocytosis and further illuminate how exocytosis is precisely regulated by calcium ions and a plethora of proteins.

## Author Contributions

RX, HM, JY, JX, ZH, and HL wrote the manuscript. All authors contributed to the article and approved the submitted version.

## Conflict of Interest

The authors declare that the research was conducted in the absence of any commercial or financial relationships that could be construed as a potential conflict of interest.

## Publisher’s Note

All claims expressed in this article are solely those of the authors and do not necessarily represent those of their affiliated organizations, or those of the publisher, the editors and the reviewers. Any product that may be evaluated in this article, or claim that may be made by its manufacturer, is not guaranteed or endorsed by the publisher.
